# A novel *Escherichia coli* cell–based bioreporter for quantification of salicylic acid in cosmetics

**DOI:** 10.1007/s00253-024-13006-8

**Published:** 2024-01-19

**Authors:** Yeonhong Kim, Yangwon Jeon, Geupil Jang, Bong-Gyu Kim, Youngdae Yoon

**Affiliations:** 1 Department of Environmental Health Science, Konkuk University, Seoul, 05029 Republic of Korea; 2 School of Biological Sciences and Technology, Chonnam National University, Gwangju, 61186 Republic of Korea

**Keywords:** Transcription factors (TFs), Multiple antibiotic resistance (Mar), Salicylic acid (SA), Bacterial cell–based bioreporters, Environmental monitoring

## Abstract

**Abstract:**

Transcription factor–based bioreporters have been extensively studied for monitoring and detecting environmental toxicants. In *Escherichia coli*, the multiple antibiotic resistance regulator (MarR) induces transcription upon binding to salicylic acid (SA). We generated SA-specific *E. coli* cell–based bioreporters utilizing the operator region of the *mar* operon and MarR as components of the reporter and sensing domains, respectively. Although bioreporters based on endogenous MarR and wild-type *E. coli* cells responded to SA, their sensitivity and selectivity were insufficient for practical sample monitoring. To improve these parameters, we genetically engineered host strains for optimal MarR expression, which enhanced the sensitivity of the biosensor to micromolar quantities of SA with increased selectivity. Under the optimized experimental conditions, the biosensor could quantify SA in environmental samples. For validation, the SA concentration in artificially contaminated SA-containing cosmetic samples was determined using the developed biosensor. Reliability assessment by comparing the concentrations determined using LC–MS/MS revealed > 90% accuracy of the bioreporters. Although bioreporters are not considered standard tools for environmental monitoring, bacterial cell–based bioreporters may serve as alternative tools owing to their affordability and simplicity. The SA biosensor developed in this study can potentially be a valuable tool for monitoring SA in environmental systems.

**Key points:**

*• SA-responsive bioreporter is generated by employing mar operon system in E. coli*

*• SA specificity and selectivity were enhanced by genetic/biochemical engineering*

*• The novel bioreporter would be valuable for SA monitoring in environmental systems*

**Supplementary Information:**

The online version contains supplementary material available at 10.1007/s00253-024-13006-8.

## Introduction

Salicylic acid (SA) is a critical plant hormone that regulates a wide variety of metabolic pathways in plants, including those for defense against abiotic and biotic stress (Delaney et al. 1994; Hara et al. 2012; Yuan and Lin 2008). An increase in SA levels induces metabolic pathways in plants (Khan et al. 2015; Wani et al. 2017), especially tolerance to diverse abiotic stress factors. SA affects growth, photosynthesis, and carbohydrate metabolism in maize plants under salt stress (Khodary 2004) and induces physiological and biochemical changes in wheat seedlings under water stress (Singh and Usha 2003). It plays a crucial role in the response to abiotic stresses across various plant species (Khan et al. 2015; Pál et al. 2013).

In addition to its role as a plant hormone, SA and its derivative, aspirin (acetylsalicylic acid), have various applications in different industries. SA is an active ingredient in various medications and personal care products. Moreover, it is used as a food preservative and a plant growth regulator to enhance productivity in the agriculture industry. Owing to its widespread use, SA is found in diverse products, such as foods, medicines, cosmetics, and preservatives (Ekinci et al. 2011; Ikarashi et al. 2010). SA can potentially have a negative impact on the environment (Richardson et al. 2007). Even at low concentrations, SA can be harmful to fish, amphibians, and other aquatic organisms, affecting their growth and reproduction (Fent et al. 2006; Nunes et al. 2015). Due to the extensive utilization of SA in various industries, it is possible that SA is present in influent/effluent wastewater and sludge at relatively higher concentrations than other pharmaceuticals and anti-inflammatory drugs. Moreover, the accumulation of SA in soil and water can lead to the long-term exposure of organisms inhabiting these environments.

Various techniques, including instrumental analysis and optical sensors based on nanomaterials, can be used to monitor or quantify SA levels (Engelberth et al. 2003; Tabassum 2022; Wilbert et al. 1998). In addition, aptamer- and fluorescence-based sensors for SA have been reported (Chen et al. 2019; Mishra et al. 2004). Although SA quantification using analytical instruments and recently developed techniques is accurate and precise, it is limited by access to expensive tools and novel techniques. To overcome these disadvantages, bacterial cell–based bioreporters have been suggested as alternatives to conventional instrumental analyses. Bacterial cell–based bioreporters have been extensively studied owing to their simplicity and low cost (Banerjee and Bhunia 2010; Gupta et al. 2019). Most bacterial cell–based bioreporters are based on stress-responsive genetic systems in microorganisms (Mahr and Frunzke 2016). To survive environmental changes, microorganisms possess defense mechanisms to detoxify external stressors, such as chemicals, heavy metals, and antibiotics. In most cases, the first step in defense involves the recognition of external stress by specific transcription factors (TFs). These proteins then trigger defense mechanisms by initiating the transcription of a series of genes or by activating specific pathways. Based on this concept, a wide variety of bacterial cell–based bioreporters for heavy metals, antibiotics, and harmful chemicals have been developed (Fernandez-López et al. 2015; Mulchandani 2011; Vogrinc et al. 2015). SA-sensing bioreporters can be constructed using genetic systems that regulate certain defense mechanisms against SA exposure.

The mar operon, consisting of multiple antibiotic resistance regulator (MarR), MarA, and MarB, is a multidrug resistance genetic system in *Escherichia coli* (Cohen et al. 1993; Seoane and Levy 1995). The expression of genes of this operon is regulated by MarR upon interaction with SA. The mode of MarR and SA binding was identified using X-ray crystallography (Alekshun et al. 2001). Recently, Zou et al. reported an *E. coli* cell–based bioreporter for SA using MarR-P_*marO*_ (Zou et al. 2021). They demonstrated the tunability of the SA response and detection ranges of the bioreporter by modulating the interaction between DNA and MarR. Nevertheless, it is crucial to conduct further investigations on the practical applications of SA bioreporters. In this regard, we further investigated the SA bioreporter using MarR-P_marO_ as a genetic system and enhanced its SA detection performance by genetically engineering MarR and *E. coli* strains. Under optimized experimental conditions, the developed bioreporter could detect SA at micromolar concentrations. In addition, the bioreporter could be used in determining SA concentrations in artificially contaminated samples originating from commercially available cosmetics. The determined concentrations were comparable to those estimated using the LC–MS/MS analysis, indicating that the developed SA bioreporter could be reliably used to quantify SA released in environmental systems.

## Materials and methods

### Materials

*E. coli* DH5α and *E. coli* BL21 were used as competent cells for gene cloning and as host cells for bioreporters, respectively. A Quick & Easy *E. coli* Gene Deletion Kit (Gene Bridges, Heidelberg, Germany) was used to delete endogenous genes in *E. coli*. pCDF-Duet and pET-21(a) were used to construct bioreporter plasmids carrying the sensing and reporter elements, respectively. HotStar Taq and Pfu Turbo (Qiagen, Hilden, Germany) were used for DNA amplification and site-directed mutagenesis, respectively. Restriction enzymes and T4 DNA ligase were purchased from Takara Bio Inc. (Shiga, Japan). The chemicals, including SA, *p*-coumaric acid, caffeic acid, ferulic acid, methyl salicylate, and aspirin, were purchased from Sigma-Aldrich (MO, USA). Primers used in this study were purchased from Macrogen (Seoul, Korea). The SA-containing cosmetics were purchased from Korean cosmetic companies.

### Plasmid construction

The gene encoding MarR was amplified using polymerase chain reaction (PCR) with primers corresponding to the genomic DNA of *E. coli* and cloned into pCDF-Duet at *Nco*I and *Not*I sites to construct pCDF-MarR. The operator region of mar operon, *P*_*marO*_, was amplified from the *E. coli* genomic DNA and inserted into pZnt-eGFP (Yoon et al. 2016) at *Bgl*II and *Xba*I sites to generate pMarO-eGFP. Besides MarR WT, diverse mutants were generated to modulate the sensitivity and selectivity of bioreporter. Based on the analysis of the 3-dimensional structure of MarR, the amino acids around SA binding sites were selected and mutated (PDB ID No. 5H3R). The mutations were generated by the site-directed mutagenesis using a pair of primers. The amino acids, Thr72 and Arg86, were selected and mutated to generate MarR T72A, T72V, and R86A. To evaluate the effects of mutagenesis, MarR mutants were introduced to *marR*-deficient *E. coli* with pMarO-eGFP. The primers used for plasmid construction in this study are listed in Table 1, and the sequences of plasmids were confirmed by DNA sequencing (Macrogen, Korea).

**Table 1:** List of primers used for cloning *P*_*marO*_ and *marR*

Gene	Sequences (5′ to 3′)	Restriction enz.
*P*_*marO*_	TCGAGATCTGGTGGTTGTTATCCTGTGT CGCTCTAGAATTAGTTGCCCTGGCAAG	*Bgl*II *Xba*I
*marR*	ATATACCATGGGCGTGAAAAGTACCAGCGATC ATTATGCGGCCGCGGCCTTACGGCAGGACTTT	*Nco*I *Not*I
*marR* T72A	CTGGGAGCACTGGCCCGTATGCTGG CCAGCATACGGGCCAGTGCTCCCAG	NcoI / NotI
*marR* T72V	GACCTGGGAGCACTGGTACGTATGCTGGATCG CGATCCAGCATACGTACCAGTGCTCCCAGGTC	NcoI / NotI
*marR* R86A	GCTGGGTGGAAGCATTGCCGAACCC GGGTTCGGCAATGCTTCCACCCAGC	NcoI / NotI

The restriction enzyme recognition sequences and mutated sequences are underlined

### Generation of *E. coli* mutant strains and bioreporters

*marR*-, *marR/marA*-, and *marR/marA/marB*-deficient *E. coli* BL21 cells were generated using the Quick & Easy *E. coli* Gene Deletion Kit. The target genes, *marR*, *marA*, and *marB*, in the mar operon and the FRT-flanked PGK-gb2-neo cassette were amplified using PCR and were introduced into *E. coli* cells harboring pRedET plasmid using the Eppendorf 2510 electroporator at 1350 V, 10 μF, and 600 Ω. The cells were then induced with 10% arabinose to replace target genes with the kanamycin resistance gene (*kan*). Deletion of endogenous genes in *E. coli* was confirmed using PCR. The gene-deficient *E. coli* strains were named *E. coli-∆marR, E. coli-∆marR/∆marA*, and *E. coli-∆marR/∆marA/∆marB*. The genetic characteristics of these strains are listed in Table 2. *E. coli* cell–based bioreporters were generated by introducing the sensing and reporter plasmids, pCDF-MarR and pMarO-eGFP, respectively, into the wild-type (WT) and mutant *E. coli* strains either alone or in combination. The characteristics of the bioreporters investigated in this study are listed in Table 2.

**Table 2:** List of *Escherichia coli* strains, plasmids, and biosensors used in this study

	Name	Genetic properties	Ref
Strains	*E. coli* BL21 (DE3)	F^−^ ompT hsdS_B_(r_B_^−^m_B_^−^)gal dcm lon (DE3)	Stratagene
	*E. coli-∆marR*	*marR-*deficient *E. coli* (*marR* is replaced by *kan*^*R*^)	This study
	*E. coli-∆marR/∆marA*	*marR/marA*-deficient *E.coli*	This study
	*E. coli-∆marR/∆marA/∆marB*	*marR/marA/marB*-deficient *E. coli*	This study
Plasmids	pET-21(a)	pBR322 ori, Amp^r^	Novagene
	pCDF-Duet	CloDE13 ori, Str^r^	Novagene
	pMarO-eGFP	pET-21(a) carrying the operator region of *marR* (MarO) in *E. coli* and *egfp* from pEGFP-N1	This study
	pCDF-MarR	pCDF-Duet carrying *marR* from *E. coli*	This study
Bioreporters	SA 1	*E. coli* BL21 (DE3) harboring pMarO-eGFP	This study
	SA 2	*E. coli* BL21 (DE3) harboring pMarO-eGFP and pCDF-MarR	This study
	SA 3	*E. coli-∆marR* harboring pMarO-eGFP	This study
	SA 4	*E. coli-∆marR* harboring pMarO-eGFP and pCDF-MarR	This study
	SA-T72A	SA3 harboring pCDF-MarR-T72A	This study
	SA-T72V	SA3 harboring pCDF-MarR-T72V	This study
	SA-R86A	SA3 harboring pCDF-MarR-R86A	This study

### Bioreporter assays

#### Selectivity test

A bioreporter assay was performed to verify the sensitivity of the bioreporters, following a previously reported protocol with minor modifications (Jeon et al. 2022). In brief, the bioreporter cells were grown overnight at 37 °C; the culture was then inoculated in fresh Luria–Bertani medium. The cells were grown for approximately 2 h until the optical density at 600 nm (OD_600_) was 0.3–0.5. The cells were then exposed to different concentrations of chemicals, including SA, and collected after 2 h of incubation. They were resuspended in 50 mM Tris–HCl (pH 7.0), and the eGFP fluorescence signals were measured using a fluorescence spectrophotometer.

#### Effects of MarR

To verify the role of MarR in the mar operon, the responses of the bioreporters, namely, SA1, SA2, SA3, and SA4, to SA were compared. Similar to the procedure described in the “Selectivity test” section, the bioreporters were exposed to 1 mM SA, and the eGFP fluorescence from each bioreporter was measured by the fluorescence spectrophotometer.

#### Effects of MarR overexpression

To validate the effect of the amount of MarR on the performance of the bioreporters, the expression levels of recombinant *marR* were modulated by induction with isopropylthio-*β*-galactoside (IPTG). The bioreporters were induced with 0–50 μM of IPTG at an OD_600_ of 0.2 and subsequently exposed to 1 mM of SA at an OD_600_ of 0.4. The expression levels of eGFP were analyzed after 2 h of exposure and indicated as induction coefficients.

#### Effects of mutations on MarR

To evaluate the effects of MarR mutants on the selectivity and sensitivity of bioreporters, MarR mutants were introduced to *marR*-deficient *E. coli* with pMarO-eGFP. The bioreporters using different MarRs as sensing elements were applied to the bioreporter assay to investigate the effects of mutations on target selectivity and sensitivity. The bioreporters were exposed to SA and its derivatives, and the fluorescence signals were measured and compared to the bioreporter having MarR WT as the sensing element.

### Measurement of fluorescence signals from the bioreporters

*E. coli* cells (1 mL) exposed to SA and its derivatives were collected, and the cell pellets were resuspended in 50 mM Tris–HCl (pH 7.4). The samples were then placed in a fluorescence spectrophotometer (FluoroMate FS-2; Scinco, Korea) equipped with a xenon lamp as a light source to measure the intensity of eGFP fluorescence. The excitation wavelength was fixed at 480 nm, and emission fluorescence signals were collected at 500–600 nm ranges with a bandwidth filter of 5 nm. The arbitrary unit for fluorescence signals was converted to the induction coefficient (fluorescence signals with exposure/fluorescence signals without exposure) after correcting for OD_600_ values. The emission fluorescence values at 520 nm were taken for the analysis.

### Quantification of SA in commercial cosmetics

#### Bioreporter-based assay

To evaluate the applicability of the newly developed bioreporters, SA levels in artificially contaminated samples were quantified. The samples were prepared by mixing different concentrations of commercial cosmetics with water. As described in the above section, the samples were added to the bioreporters SA2 and SA4 together with 10 μM IPTG. The eGFP fluorescence intensities for the bioreporters were determined, and the SA concentrations were calculated based on standard curves. A standard curve was obtained by spiking with a known concentration of SA, ranging from 0 to 5 mM under the same experimental conditions.

#### Quantification using LC–MS/MS

The SA concentrations in the cosmetic samples were quantified using an LCMS-8060 equipped with a Nexera X2 UHPLC (Shimadzu Corporation, Japan) coupled with an Acquity UPLC BEH Shield RP18 column (150 × 2.1 mm, 1.7 µm, Nihon Waters K. K., Japan). The cosmetic samples were serially diluted up to 20,000 times and injected. Separation was performed using gradient programs with 0.1% formic acid in water as mobile phase A and 0.1% formic acid in acetonitrile as mobile phase B; the flow rate was 0.2 mL/min. For the subsequent MS/MS analysis, nitrogen and argon gases were used as the heating and drying and collision gases, respectively. SA was detected in multiple reaction monitoring mode at *m*/*z* 137.2–93.0, 137.2–65.1, and 137.2–74.9 as product ions with corresponding collision energies of 15, 34, and 25 V, respectively.

### Statistical analysis

Statistical analysis and data validation were performed using R version 4.3.0 and package DescTools version 0.99.59 (Signorell et al. 2019; Team RDC 2009).

## Results

### Specificity of bioreporters toward salicylic acid

**Fig. 1:**
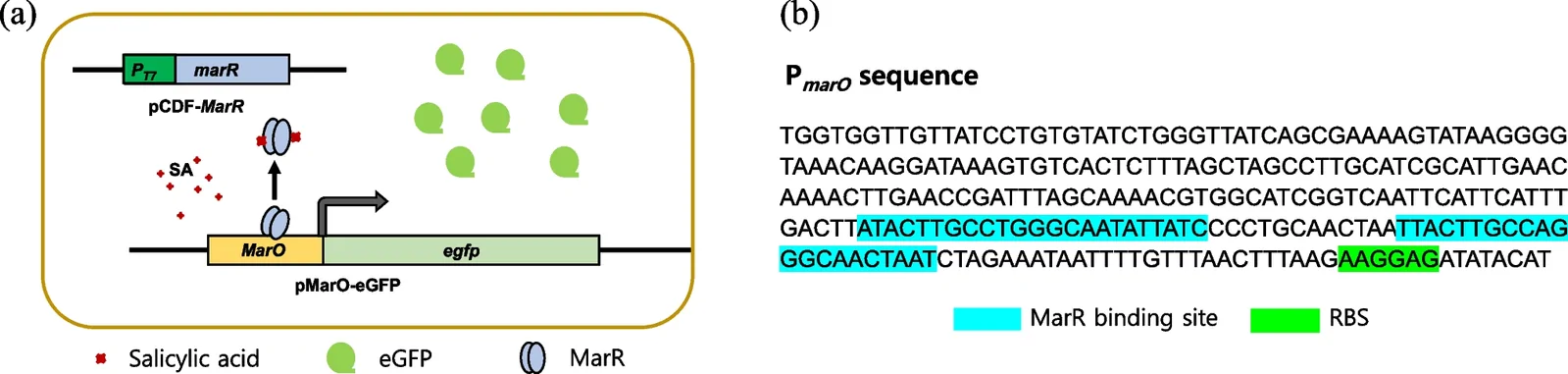
Illustration of salicylic acid (SA) biosensors. **a** The working mechanism of SA-sensing biosensors. The *egfp* fused with the promoter region (*P*_*marO*_) of the mar operon was regulated by MarR in the presence of SA. SA induced the release of MarR from the promoter region, resulting in the expression of eGFP. **b** The DNA sequence of *P*_*marO*_ used for constructing the SA-sensing plasmid. The highlighted regions indicate the MarR-binding and ribosome-binding sites

**Fig. 2:**
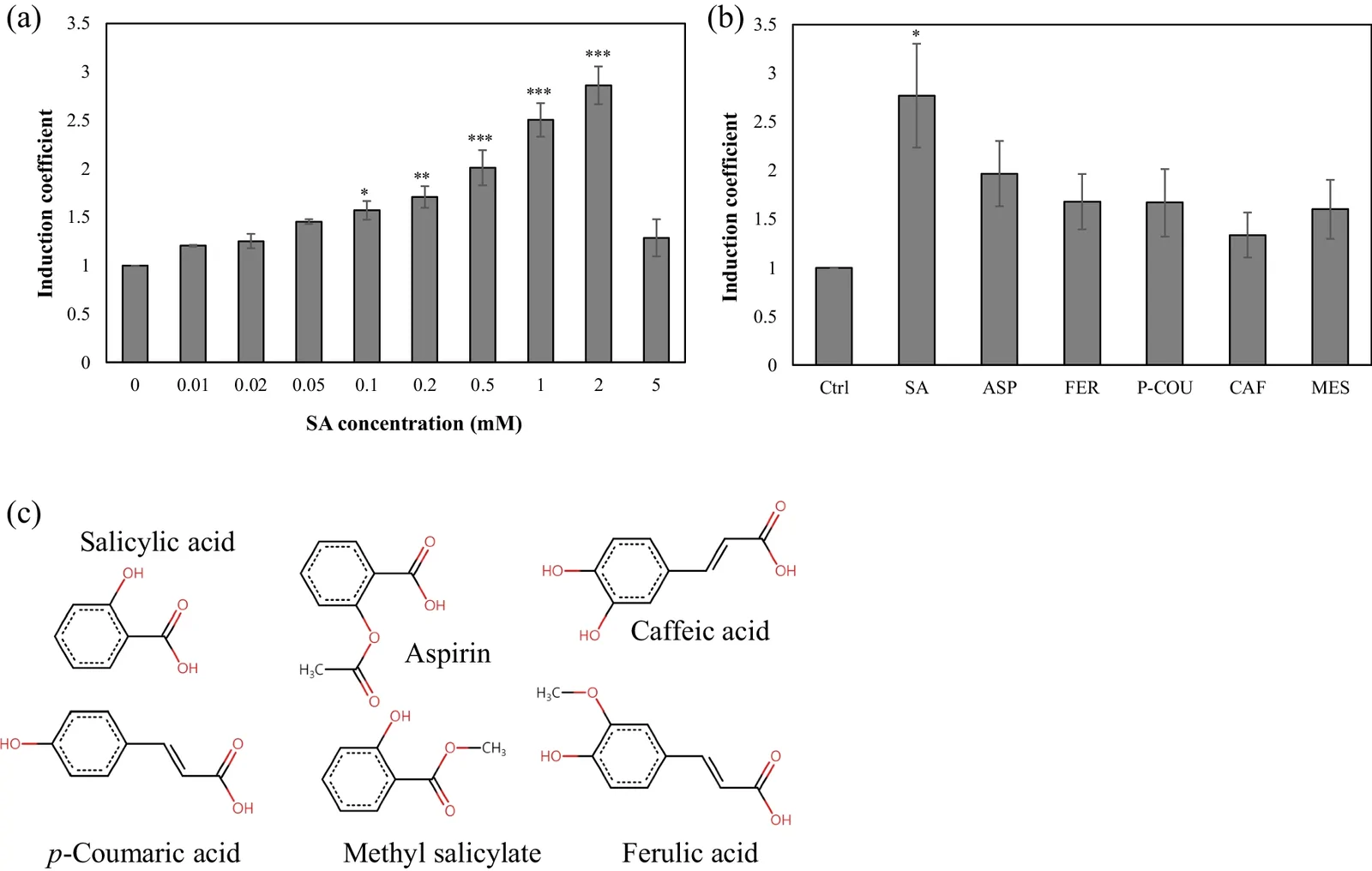
Responses of biosensors based on *Escherichia coli* BL21 WT harboring pMarO-eGFP toward salicylic acid (SA) and other compounds. **a** Responses of biosensors toward SA (0–5 mM) exposure. The biosensors were exposed to SA for 2 h, and the green fluorescence signals were converted to induction coefficients. **b** Responses of biosensors toward 1 mM of SA and other derivatives (Ctrl, without exposure; SA, salicylic acid; ASP, aspirin; FER, ferulic acid; P-COU, *p*-coumaric acid; CAF, caffeic acid; MES, methyl salicylic acid). **c** Chemical structures of SA and its derivatives. Asterisks indicate significant difference in data compared with control (*P ≤ 0.05, **P ≤ 0.01, ***P ≤ 0.001 using Dunnett’s test)

The SA-responsive bioreporters were constructed by introducing the plasmid pMarO-eGFP carrying *P*_*marO*_*::egfp* into *E. coli* BL21 WT. As shown in Fig. 1a, the transcription of *egfp* under *P*_*marO*_ was regulated by endogenous MarR, and the presence of SA induced the release of MarR from the operator sequence, resulting in the expression of eGFP. Thus, the amount of SA was determined by measuring the eGFP expression. The MarR-binding sites in the operator region are shown in Fig. 1b. To elucidate the SA-sensing ability of the bioreporter, a bioreporter assay was conducted using 0–5 mM SA. After 2 h of exposure, the intensity of eGFP fluorescence was measured and indicated as an induction coefficient (Fig. 2a). The response toward SA increased in a concentration-dependent manner; however, the signal started to decrease above 2 mM SA. This could be due to the toxic effects of SA on *E. coli* growth. Although the bioreporter showed proportional responses toward SA, the induction coefficients upon SA exposure were not sufficient for quantification because only a 3-fold increase was observed at 2 mM SA exposure. Thus, it was necessary to improve the sensitivity of these bioreporters for use as a tool to monitor SA.

In addition to sensitivity, the selectivity toward targets is another critical aspect of bioreporters. To verify selectivity, the bioreporters were exposed to 1 mM SA and its derivatives, including aspirin, ferulic acid,* p*-coumaric acid, caffeic acid, and methyl salicylic acid (Fig. 2b). Although the best response was obtained upon SA treatment, weak responses with induction coefficients < 2 were also obtained from the tested derivatives. Because all the tested compounds were similar in structure, it would be desirable that the bioreporters exhibit broad selectivity (Fig. 2c). Notably, the fluorescence intensity of eGFP without exposure was > 3000 arbitrary units (Figure S1). It was assumed that the ability of endogenous MarR to act as a repressor was insufficient to repress the transcription of genes under *P*_*marO*_. To clarify this, the sensitivity of the bioreporters to SA was further investigated with different levels of MarR expression.

### The roles of MarR on SA-sensing properties

**Fig. 3:**
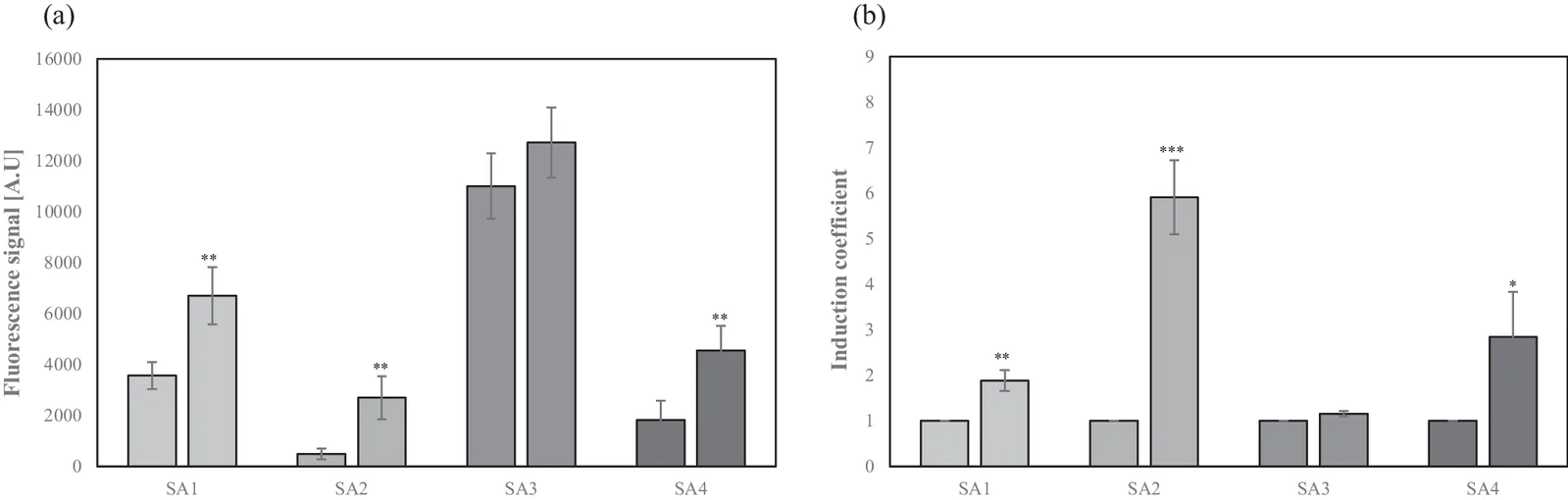
Responses of genetically different biosensors to 1 mM salicylic acid (SA). **a** The arbitrary units of eGFP induced by 1 mM SA exposure in the four types of *Escherichia coli* cell–based biosensors. **b** The eGFP signals were converted to induction coefficients (SA1, *E. coli* BL21 wild-type (WT) harboring pMarO-eGFP; SA2, *E. coli* BL21 WT harboring pMarO-eGFP and pCDF-MarR; SA3, *marR*-deficient *E. coli* BL21 harboring pMarO-eGFP; SA4, *marR*-deficient *E. coli* BL21 harboring pMarO-eGFP and pCDF-MarR). Asterisks indicate significant difference in data compared with control (*P ≤ 0.05, **P ≤ 0.01, ***P ≤ 0.001 using the two-tailed Student’s *t*-test)

Since MarR acts as a target sensing module and is a regulator in the *mar* operon, it was necessary to elucidate its role in this bioreporter system. As *E. coli* BL21 WT possessing endogenous MarR was used as the host strain for the bioreporters, a *marR*-deficient *E. coli* strain (*E. coli-∆marR*) was generated and used as a host strain for comparison. To investigate the role of MarR in bioreporters, all four bioreporters developed in this study were used in the bioreporter assay. The bioreporters were exposed to 1 mM SA at an OD_600_ of 0.3–0.4, and the fluorescence signals were measured after 2 h (Fig. 3). As shown in Fig. 3a, the fluorescence signals from all the bioreporters, except for SA3, were lower without SA exposure than they were after the exposure. For SA3, strong fluorescence signals were observed regardless of SA treatment. It was assumed that MarR acts as a repressor and that the genes under *P*_*marO*_ are transcribed constitutively without MarR. However, SA4, which was SA3 with recombinant MarR, showed responses similar to those of SA1 and SA2. These results confirmed that the mar operon was regulated by MarR as a transcriptional regulator and that MarR was responsible for SA-related responses. Interestingly, the repression by recombinant MarR was higher than that by endogenous MarR when comparing SA1 and SA4. However, this was not caused by functional differences in endogenous and recombinant MarRs because the amino acid sequences of both endogenous and recombinant MarRs were identical. This was due to the amount of MarR expressed by the recombinant and endogenous genes. This was confirmed by comparing the responses of SA1 and SA2 without SA exposure (Fig. 3a). SA2, with both endogenous and recombinant MarR, showed a much weaker signal and, thereby, had the highest induction coefficient (Fig. 3b). This answers the question discussed in the “Specificity of bioreporters toward salicylic acid” section on how the amount of MarR was related to the sensitivity of bioreporters. Thus, bioreporter sensitivity can be modulated by controlling the expression of regulatory proteins.

### Effect of MarR overexpression on bioreporters

**Fig. 4:**
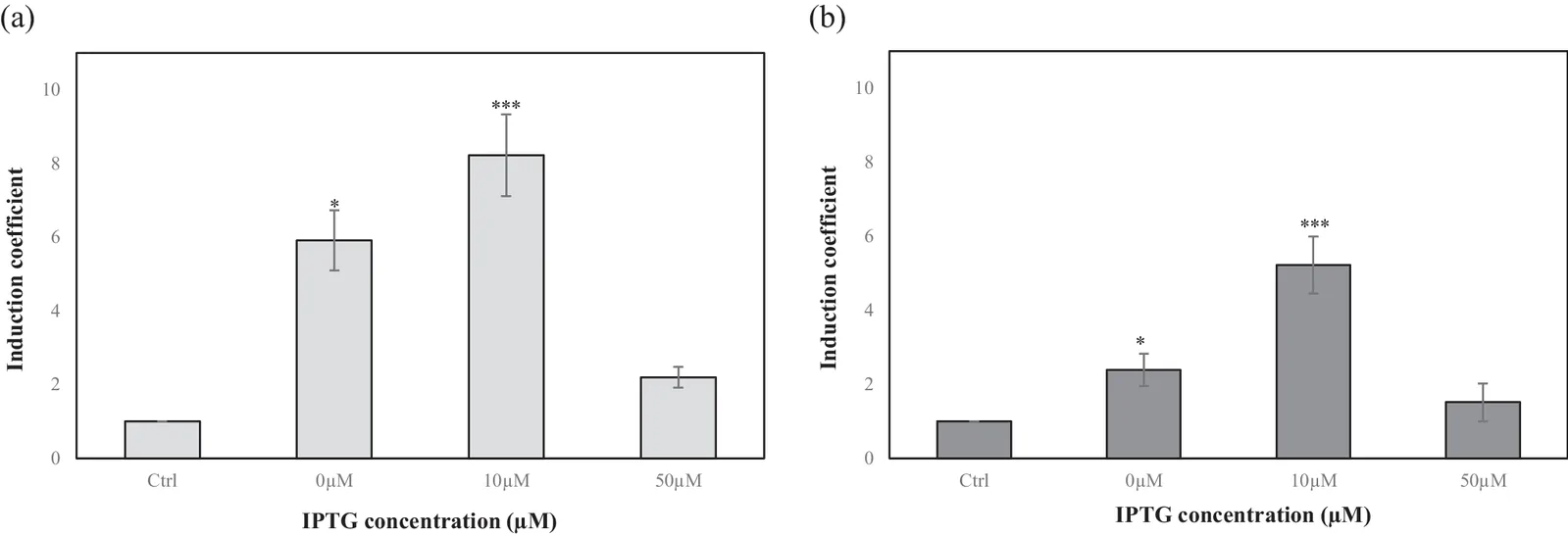
Effect of MarR overexpression by induction with isopropylthio-*β*-galactoside (IPTG) on *Escherichia coli* cell–based biosensors. The induction coefficients of biosensors SA2 (**a**) and SA4 (**b**) exposed to 1 mM of salicylic acid (SA) after induction with different concentrations of IPTG. Control indicates the biosensor without SA exposure. The data were derived from more than three replicate experiments. Asterisks indicate significant difference in data compared with control (*P ≤ 0.05, **P ≤ 0.01, ***P ≤ 0.001 using Dunnett’s test)

The sensitivity of SA bioreporters was enhanced by increasing the level of MarR in *E. coli* cells. The induction coefficients of the bioreporters to 1 mM SA increased approximately 3-fold upon the addition of recombinant *marR* located under the T7 promoter in the pCDF-Duet vector. Because the sensitivity of the SA bioreporter was related to the amount of MarR, the expression level of MarR was modulated using 0, 10, and 50 μM IPTG. Among the tested concentrations, 50 μM IPTG inhibited the growth of *E. coli* resulting in a decrease in induction coefficients (Fig. 4). At 10 μM, IPTG treatment resulted in approximately 20% and 40% increase in signals from SA2 and SA4, respectively, in response to 1 mM SA (Fig. 4). The increase in MarR expression enhanced the response to SA with respect to the induction coefficient by lowering the signals (arbitrary fluorescence signals) without SA treatment. Thus, it was concluded that the bioreporter SA2 with 10 μM IPTG treatment was optimal for monitoring SA, and this experimental condition was used for further study.

### Target selectivity of *E. coli* cell–based bioreporters

**Fig. 5:**
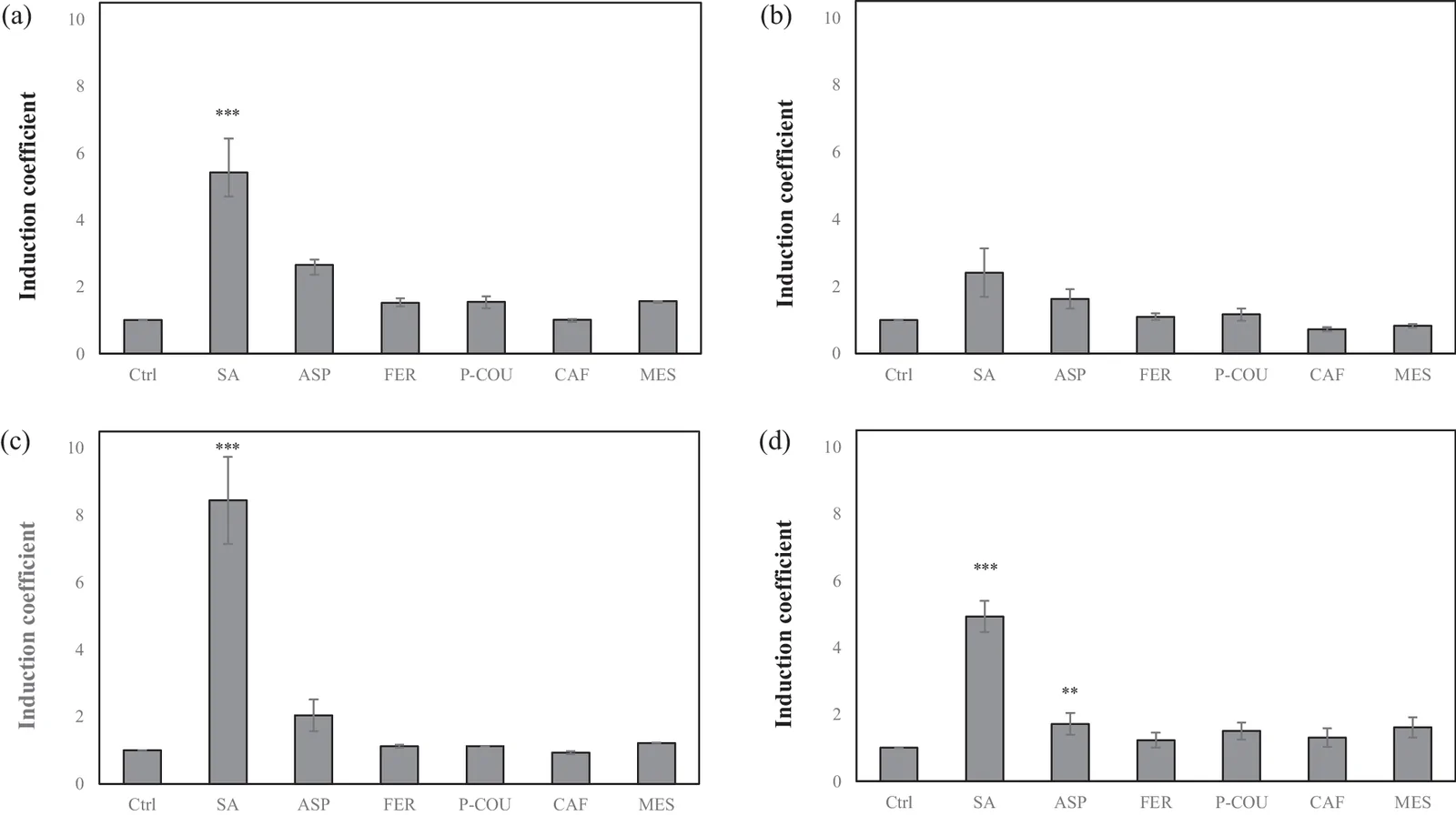
Effect of MarR overexpression on the sensitivity and selectivity of biosensors toward salicylic acid. **a** Responses of the biosensor SA2 to 1 mM SA and its derivatives without isopropylthio-*β*-galactoside (IPTG) treatment. **b** Responses of the biosensor SA4 to 1 mM SA and derivatives without IPTG. **c** Responses of the biosensor SA2 treated with IPTG. **d** Responses of the biosensor SA4 treated with IPTG (Ctrl, without exposure; SA, salicylic acid; ASP, aspirin; FER, ferulic acid; P-COU, *p*-coumaric acid; CAF, caffeic acid; MES, methyl salicylic acid). Asterisks indicate significant differences in data compared with control (*P ≤ 0.05, **P ≤ 0.01, ***P ≤ 0.001 using Dunnett’s test)

The bioreporter with both endogenous and recombinant MarR showed an improved SA response, which was enhanced by IPTG treatment. Under these experimental conditions, the target selectivity of bioreporters was further investigated because SA1, the bioreporter regulated by only endogenous MarR, *E. coli* with pMarO-eGFP, showed broad target selectivity (Fig. 2b). As shown in Fig. 5, SA2, *E. coli* with pMarO-eGFP/pCDF-MarR, and SA4, *E. coli-∆marR* with pMarO-eGFP/pCDF-MarR, were exposed to 1 mM SA and its derivatives with and without 10 μM IPTG induction. Without IPTG induction, SA2 showed approximately two times higher response toward SA than toward other chemicals, and SA4 showed a similar response as SA1 (Fig. 5a and b). In contrast, SA2 with IPTG induction showed an approximately fourfold enhanced response to SA than others, and SA4 showed a 2.5-fold enhanced response (Fig. 5c and d). As shown in Fig. 5c, the responses toward other chemicals indicated about 1–2 times the induction of coefficients, whereas the response toward SA was 8.5 times the induction of coefficients. Thus, the increase in MarR expression in the bioreporters improved the selectivity and sensitivity only toward SA, whereas the responses toward other tested chemicals were not affected by IPTG treatment. Although the MarR levels in *E. coli* cells were not determined, and interference caused by other factors should be considered, it was concluded that the bioreporter SA2 treated with IPTG was an SA-specific bioreporter.

### Effects of MarR mutants on bioreporters

**Fig. 6:**
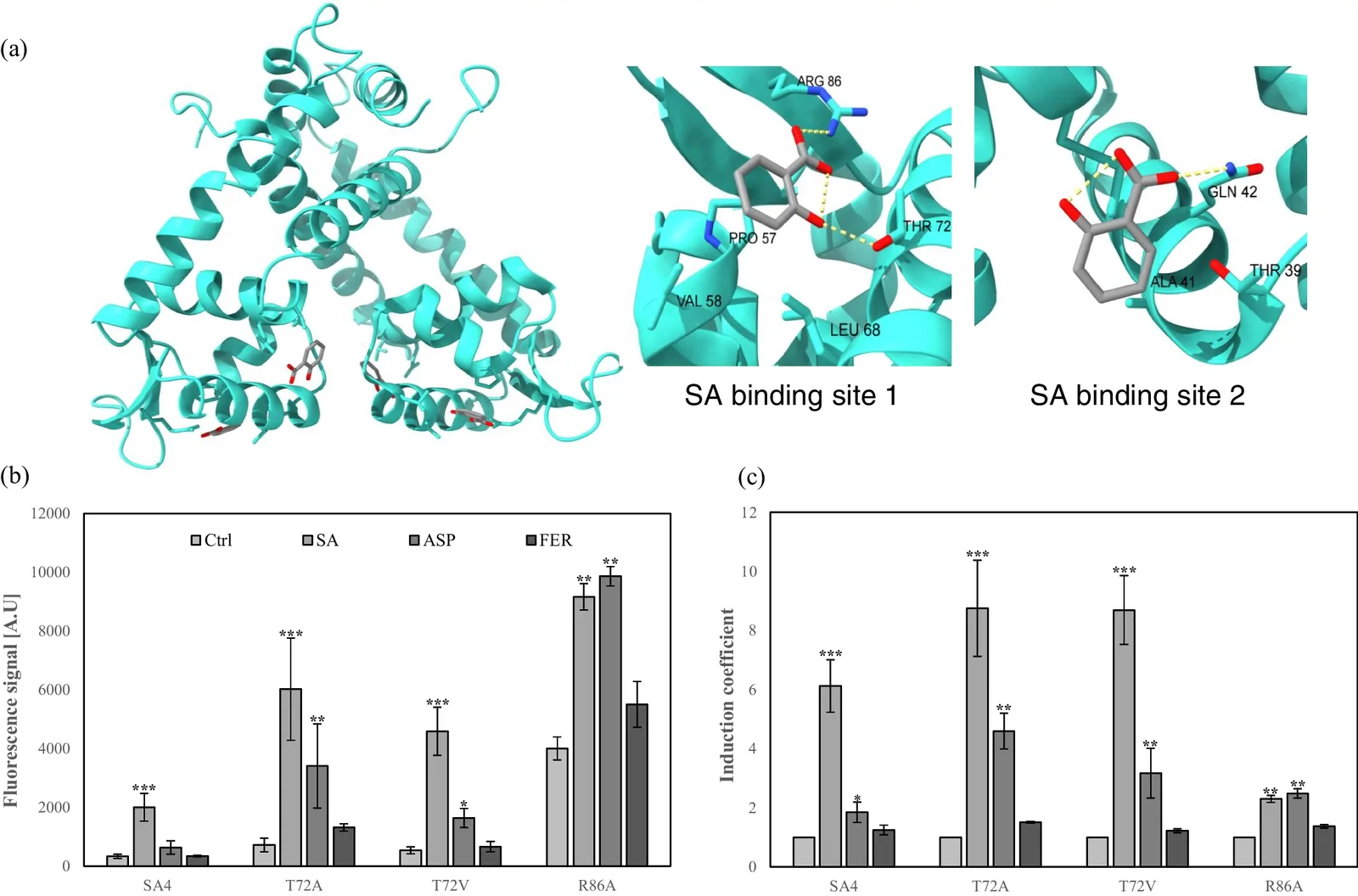
The structure of MarR dimer and the responses of bioreporter employing MarR mutants as sensing elements to SA and its derivatives. **a** A dimer structure of MarR with 4 SAs (PDB ID. 5H3R). **b** The nascent fluorescence signals of SA4, T72A, T72V, and R86A induced by SA, ASP, and FER exposures. **c** The induction coefficient values of SA4, T72A, T72V, and R86A toward SA, ASP, and FER. Asterisks indicate significant differences in data compared with control (*P ≤ 0.05, **P ≤ 0.01, ***P ≤ 0.001 using Dunnett’s test)

Since the properties of TF-based bioreporters were determined by the target selectivity and specificity of TFs, the performance of bioreporters could be modulated by genetic engineering on TFs. As shown in Fig. 6a, MarR forms a dimer to play as a TF and has two SA binding sites on each monomer (Hao et al. 2014). To modulate the target sensing properties, the amino acids in SA binding sites 1 and 2 were mutated to generate 2 MarR mutants named MarR-T72A, MarR-T72V, and MarR-R86A. They were introduced in *E. coli-∆marR* with pMarO-eGFP and compared to SA4. The responses toward SA and its derivatives were measured by the bioreporter assay. As a result, it was noticed that the responses toward SA and other chemicals were enhanced with the increase of background signals compared to SA4 (Fig. 6b). Although the nascent signals to SA increased, the responses from other chemicals were also increased, especially to aspirin (Fig. 6c). It was assumed that the mutations on Thr72 and R86 caused the increase in target accessibility and weakening DNA binding. On the other hand, MarR-R86A showed enhanced responses toward SA, aspirin, and ferulic acid with strong background signals. This result might be caused by the conformational changes in MarR-R86A weakening the DNA binding ability. Thus, it was noticed that the genetic engineering on TFs could show the effects on target selectivity and sensitivity of bioreporters as well as on DNA binding properties. Consequently, it was concluded that SA2 having both endogenous and exogenous MarR WT was the best bioreporter for SA quantification. Therefore, SA2 was used for SA quantification in further investigation in this study. However, the genetic engineering on MarR implied the possibility of extending the application of the mar operon system for detecting aspirin and other derivatives.

### Quantification of SAs originating from cosmetics

**Fig. 7:**
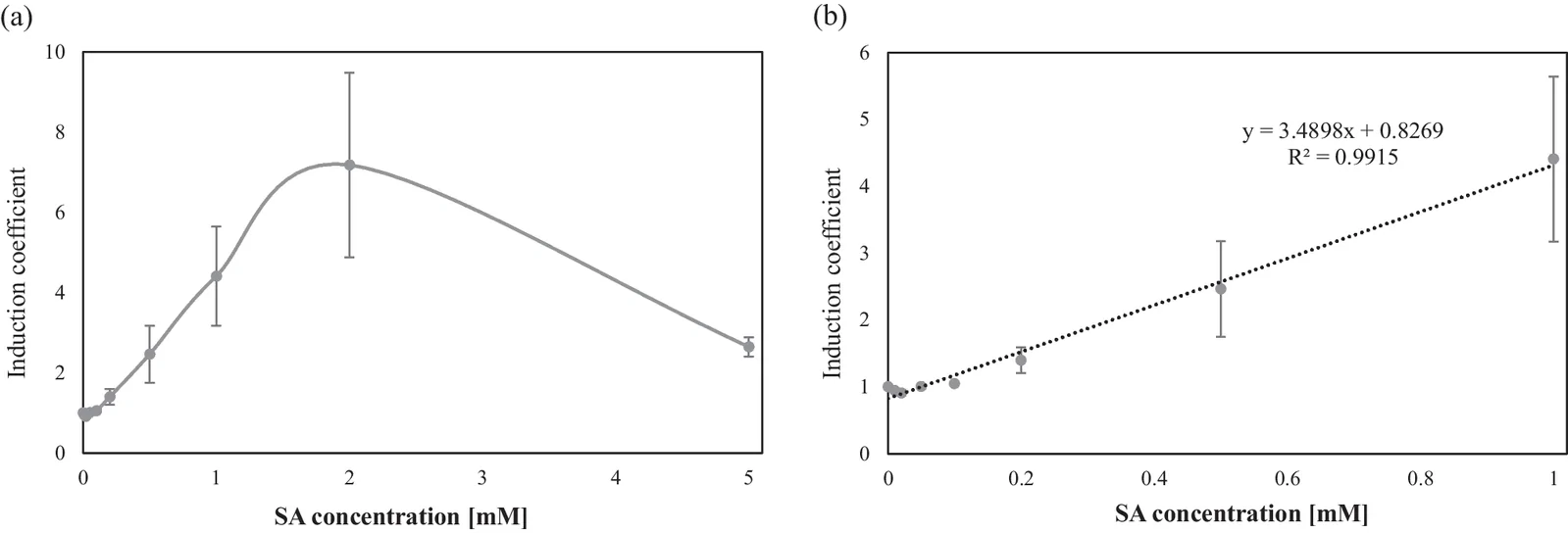
Standard curves for salicylic acid (SA) quantification. **a** Responses of biosensor SA2 exposed to 0–5 mM of salicylic acid for 2 h after 10 μM of isopropylthio-*β*-galactoside (IPTG) treatment. **b** The standard curve obtained from the biosensor SA2 for SA quantification. The data were obtained from more than three experiments. The standard deviation is indicated as error bars

To verify the applicability of the newly developed SA bioreporters, SA originating from commercially available cosmetics was quantified using the bioreporter SA2 under optimized conditions. Three different skin-soothing products were purchased and used as samples. Based on the description of ingredients, products A and B contained 0.5% and 2.0% (w/w) SA, respectively; however, no such description was available for product C. For quantification, the standard curves were obtained using the bioreporter assay for IPTG-treated SA2, and the induction coefficients were determined after 0–5 mM SA exposure (Fig. 7a). Because the responses decreased at 2 mM and high SA concentrations, a standard curve was constructed for 0–1 mM SA exposure, and the equation from linear regression analysis was used to calculate the SA concentration (Fig. 7b). Based on the calibration curve, 5 μM of SA was the lowest concentration that showed significant differences from statistical analysis, and the limit of linearity was observed at about 1.5 mM (Fig. 7).

Additionally, it was confirmed that the expression of eGFP was regulated well by MarR because the original fluorescence signals of SA2 without SA exposure showed about 200 arbitrary units (Figure S2). Various dilutions of the cosmetic samples were analyzed using the bioreporter assay under the same experimental conditions. The induction coefficients after exposure to cosmetic samples were converted to concentrations based on standard curves. The SA concentrations in products A, B, and C were calculated to be 31.55, 105.57, and 27.48 mM, respectively (Table 3). At 0.5% and 2.0% (w/w), the SA concentration in the products would be 36.2 and 145 mM, respectively. Although the values determined by the bioreporter assays were similar to those calculated from the product description, they were further confirmed using LC–MS/MS.

**Table 3:** Quantification of salicylic acid in commercial cosmetics

Cosmetics	Concentration [mM]		Accuracy (%)
	Bioreporter	LC–MS/MS	
A	31.55 ± 4.50	29.64 ± 5.07	93.9
B	105.57 ± 18.22	106.77 ± 6.26	98.9
C	27.48 ± 10.64	25.98 ± 3.24	94.5

For LC–MS/MS analysis, the 5 to 100 ppb ranges of standard SA were injected at room temperature, and the peak area was measured to obtain the calibration curve. The relationship between the concentration of SA and the peak area was analyzed by a linear regression fit and obtained a calibration curve with 0.998 *R*-squared values. The SA concentrations in the cosmetics determined using LC–MS/MS are also listed in Table 3. The performance of the bioreporter assay was represented as relative accuracy, which is defined as [values from bioreporter assay]/[values from LC–MS/MS]. The SA concentrations in products A, B, and C determined using the bioreporter assay were comparable to those determined using the LC–MS/MS analysis, showing an accuracy of > 93%. Several other ingredients present in the cosmetics might have affected the quantification using bioreporters, whereas only SA was detected using LC–MS/MS. Nonetheless, with > 93% accuracy, the SA bioreporters developed in this study showed superior SA selectivity over other bioreporters. Therefore, the SA bioreporters developed in this study can be used as an alternative tool for monitoring SA.

## Discussion

Salicylic acid is widely used in various industries, such as agriculture and pharmaceutical industries, and is an ingredient of many personal care products. Although the adverse effects of SA are not considered a severe threat, higher SA concentrations can be present in the upper trophic levels of the food chain through bioaccumulation (Bai and Acharya 2019; Boxall 2004). The instrument-based analysis was employed for SA monitoring, but it was needed to have a simple and convenient tool to monitor SA concentrations in diverse environmental systems. In this regard, bacterial cell–based bioreporters would be a good alternative as a simple and cheap technique. Except for industrial usage, SA is a plant hormone and a key factor for defense against various stresses including pathogen infections. To avoid this defense system, microorganisms such as bacteria and viruses have metabolic pathways to degrade the phytohormones. Proteins in the MarR family control multiple antibiotic resistance to antibiotics, organic solvents, and oxidative stress (Alekshun et al. 2001). These proteins also control the synthesis of pathogenic factors in some microbes that infect humans and plants (Miller and Sulavik 1996). The proteins in the MarR family have been identified in various microorganisms, and *E. coli* has MarR that regulates the mar operon negatively in the presence of SA. Thus, the operator region of mar operon (*P*_*marO*_) and MarR were employed as a genetic system for devising the SA bioreporters.

As described above, the *E. coli* having pMarO-eGFP showed responses toward SA, but the sensitivity and selectivity toward SA were not reached to the criteria for sensors. To solve this problem, the experimental conditions were optimized by genetic engineering and modulating the expression level of MarR. Conclusively, the mar operon–based bioreporters showed sufficient specificity toward SA under the optimized experimental conditions. In fact, characteristics including target specificity and selectivity, detection limitations, and detection ranges were considered for evaluating the capability of the bioreporters. Considering the working mechanisms of TF-based bioreporters, their selectivity relies on the interactions between ligands and regulatory proteins. Jiang et al. reported that the sensitivity of bioreporters for *p*-coumaric acid was modulated by controlling the strength of the promoters for regulatory proteins and by weakening the binding of regulatory proteins to the operator regions (Jiang et al. 2020). Because modulation of the strength of the promoter controls the expression levels of regulatory proteins, it is also related to the manipulation of the dynamic ranges of bioreporters as well as their sensitivity (Yoon et al. 2016; Zou et al. 2021). In this regard, it was observed the increase in MarR expression enhanced the sensitivity of SA bioreporters in this study. In addition, the genetic engineering on MarR has been investigated to enhance the performance of bioreporters. Based on structural analysis, the amino acids related to SA binding, Thr72 and Arg86, were selected and mutated. In concordance with previous studies, the mutations on MarRs showed the changes in target selectivity and sensitivity (Fig. 6). However, the bioreporters employing MarR mutants as regulatory proteins showed insufficient properties for SA qantification. Although the responses toward SA were increased, the responses from aspirin and ferrulic acid were also increased. The mutations investigated in this study might induce the conformational changes, thereby broadening target selectivity and weakening DNA binding. Because of the complexity of MarR-SA interactions, it was not easy to obtain the desired properties by simple genetic engineering. Although the enhancement of SA-sensing performance of bioreporters was not obtained by MarR engineering in this study, it was suggested the potential to extend the applications of the mar operon system for new target sensing with further investigation.

To compare the performance of the novel SA bioreporter, the *E. coli* cell–based bioreporters able to detect SA employing different TF systems are listed in Table 4. The bioreporters based on NagR and NahR from *Rarstonia* sp. and *P. putida*, respectively, showed multi-target responses because the regulatory proteins were not specific to SA even if the limit of detection (LOD) was low (Mitchell and Gu 2005; Shin 2010). Although Zou et al. had reported the SA bioreporter based on mar operon system, their invesitgation was focused on SA responses of biosensors rather than the quantitative analysis of SA. (Zou et al. 2021). On the other hand, we developed SA-specific *E. coli* cell–based bioreporters employing the mar operon system with genetic engineering and optimization of the experimental conditions because the bioreporter based on the native mar operon system showed insufficient sensitivity and selectivity toward SA. To enhance the performance of the developed bioreporters, the expression level of MarR was modulated by adding recombinant MarR and IPTG. The capability of the bioreporters to quantify SA was verified by determining the concentrations of SA originating from commercial cosmetics. Considering the high accuracy of the bioreporters in SA quantification vis-à-vis LC–MS/MS, the SA bioreporters could be used as an alternative tool to monitor SA. Our results also suggested that genetic engineering and modulation of the expression of regulatory proteins could be used to enhance the performance of bioreporters employing TF-based systems.

**Table 4:** Comparison of salicylic acid sensing *E. coli* cell–based bioreporters

Analytes	Host strains	TFs	Range of detection	Response time	Ref
SA, 3-methyl SA, Aspirin	*E. coli*	*Rarstonia sp.* NagR	2.4 ~ 2500 μM	20 min–3 h	(Mitchell and Gu 2005)
SA	*E. coli*	*E. coli* MarR	2 ~ 15 mM	Not specified	(Zou et al. 2021)
SA, naphthalene	*E. coli*	*P. putida* NahR	0.001 ~ 5 mM	2 h	(Shin 2010)
SA	*E. coli*	*E.coli* MarR	0.01 ~ 1 mM	1–2 h	This study

## Supplementary Information

Below is the link to the electronic supplementary material.

Supplementary file1 (PDF 196 KB)

## Data Availability

Not applicable.
