# Supplementary material for: A novel Escherichia coli cell–based bioreporter for quantification of salicylic acid in cosmetics
Source: Appl Microbiol Biotechnol. 2024 Jan 19;108:148. doi: 10.1007/s00253-024-13006-8 (PMC10799119; doi:10.1007/s00253-024-13006-8)

Supplementary materials for Applied Microbiology and Biotechnology titled as:

A novel *Escherichia coli* cell-based bioreporter for quantification of salicylic acid in cosmetics

Yeonhong Kim [1], Yangwon Jeon [1], Geupil Jang [2], Bong-Gyu Kim [2] and Youngdae Yoon [1,*]

[1] Department of Environmental Health Science, Konkuk University, Seoul 05029, Republic of Korea
[2] School of Biological Sciences and Technology, Chonnam National University, Gwangju 61186, Republic of Korea
[3] Division of Environmental and Forest Science, Gyeongsang National University, Jinju, 52725, Republic of Korea

***Corresponding author:**
Youngdae Yoon
Tel: +82-2-450-0443
Fax: +82-2-450-3726
E-mail: yyoon21@gmail.com

Figure S1. The responses of biosensor, *Escherichia coli* BL21 WT harboring pMarO-eGFP (SA1), toward salicylic acid. The eGFP signals were indicated as arbitrary units. Asterisks indicate significant difference in data compared with control (*$P \leq 0.05$, **$P \leq 0.01$, ***$P \leq 0.001$ using the Dunnett's test).

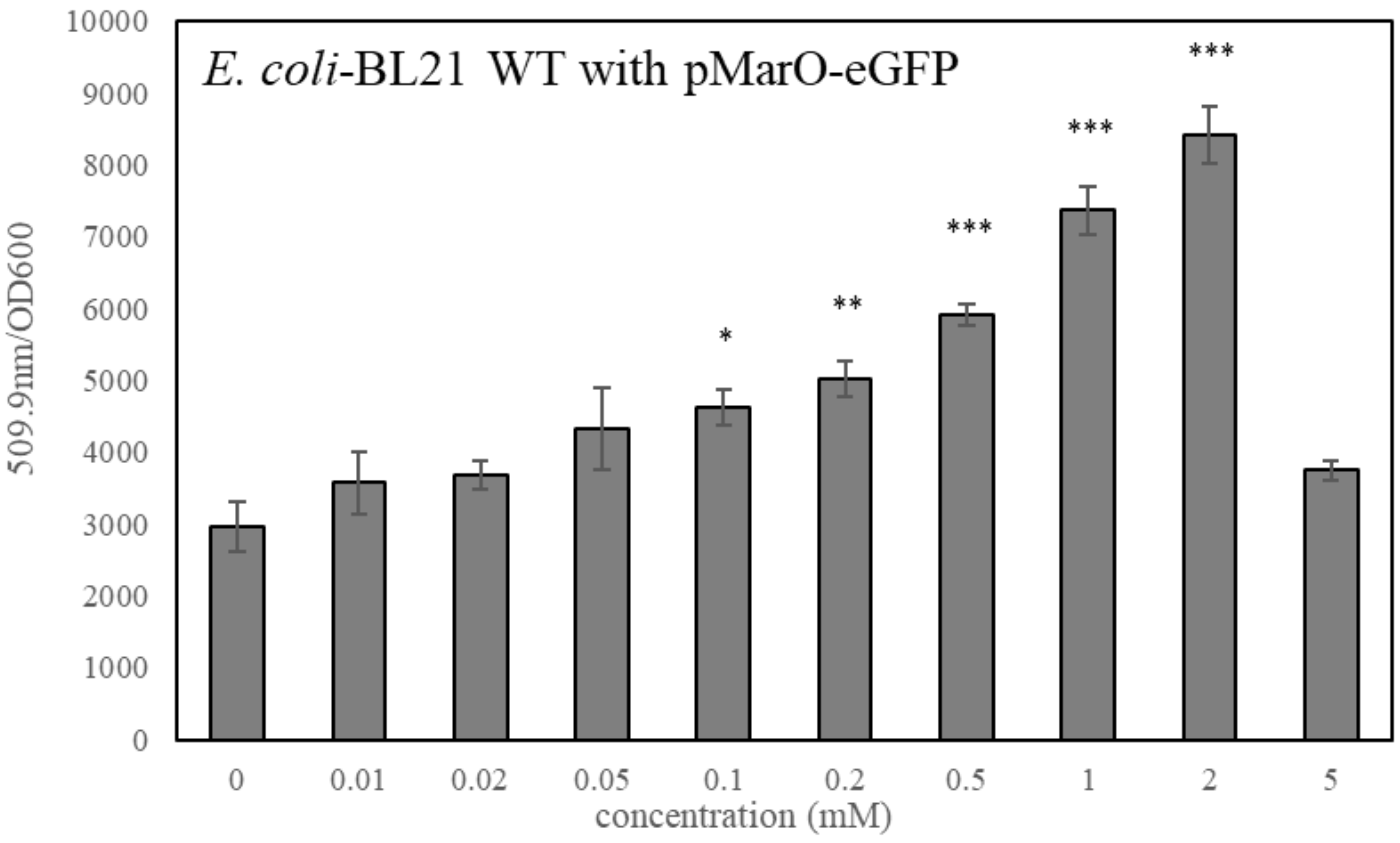

Figure S2. The fluorescence signals of standard curve indicated as arbitrary units. Responses of biosensor SA2 exposed to 0–5 mM of salicylic acid for 2 h after 10 μM of IPTG treatment. The data were obtained from more than three experiments. The standard deviation is indicated as error bars.

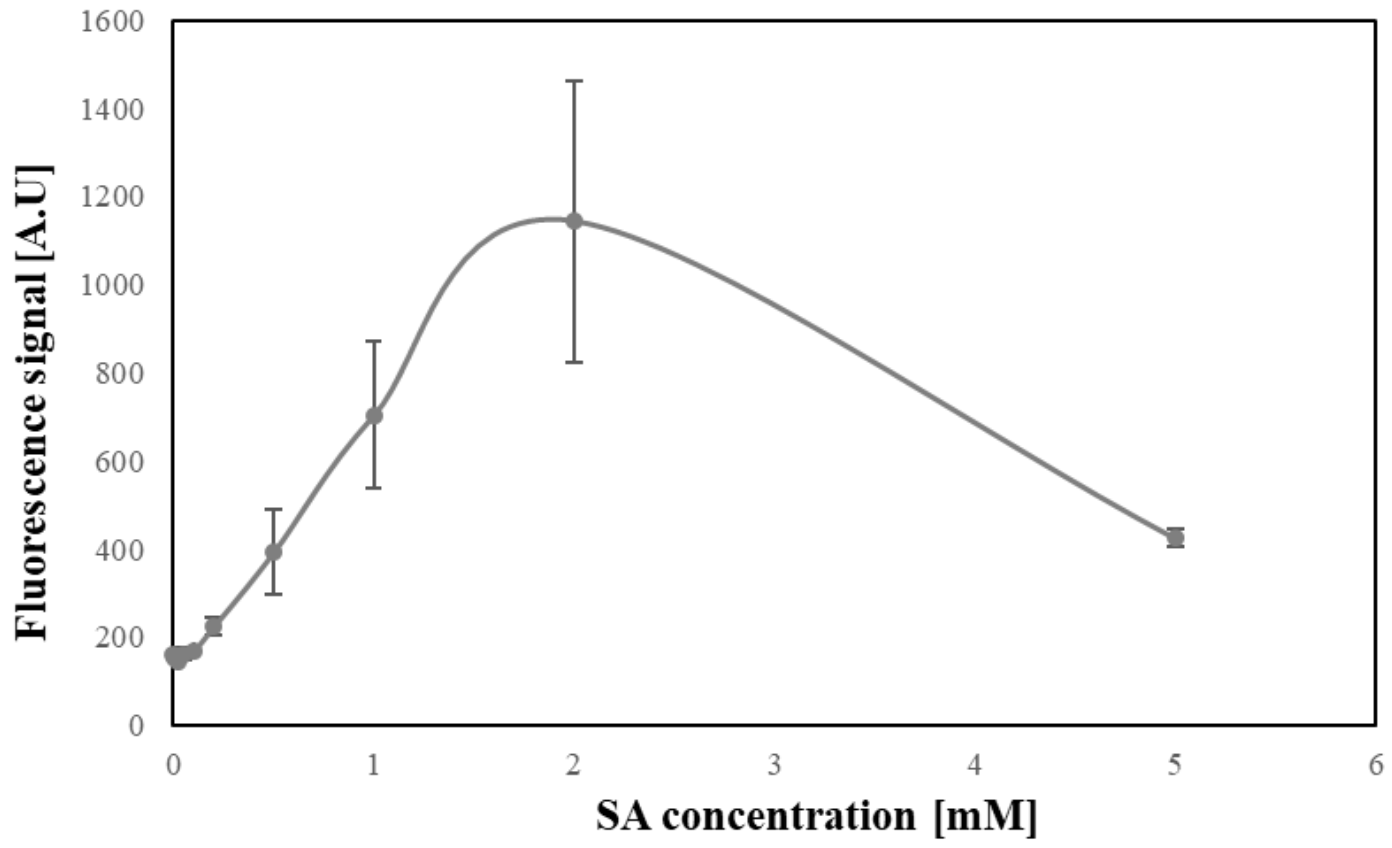

Supplement: Supplementary file 1 [file 253_2024_13006_MOESM1_ESM.pdf]
